# Molecular diagnosis of patients with congenital adrenal hyperplasia due to 21-hydroxylase deficiency

**DOI:** 10.1186/s12902-020-00643-z

**Published:** 2020-11-09

**Authors:** Tania Mayvel Espinosa Reyes, Teresa Collazo Mesa, Paulina Arasely Lantigua Cruz, Adriana Agramonte Machado, Emma Domínguez Alonso, Henrik Falhammar

**Affiliations:** 1 National Institute of Endocrinology, Zapata Street and D, Vedado, 10400 Havana, Cuba; 2 National Center for Medical Genetics, Havana, Cuba; 3 Department of Molecular Medicine and Surgery, Karolinska Institutet, Stockholm, Sweden; 4 Department of Endocrinology, Metabolism and Diabetes, Karolinska University Hospital, Stockholm, Sweden

**Keywords:** Genetics, Point mutations, Congenital adrenal hyperplasia

## Abstract

**Background:**

Congenital adrenal hyperplasia (CAH) is an autosomal recessive group of diseases. 21-Hydroxylase deficiency (21OHD) accounts for between 95 and 99% of all CAH cases.

**Objectives:**

To characterize the genotype of patients clinically diagnosed with 21OHD and to identify the most frequent mutations in the Cuban population.

**Methods:**

Cross-sectional descriptive study that included all patients diagnosed with 21OHD from January 2000 to December 2018. For the molecular analysis of the *CYP21A2* gene, a protocol was used that used the polymerase chain reaction in 2 stages; in the first stage genomic DNA was amplified and 5 point mutations were detected in the second stage (Intron 2, Deletion of 8 bp, G318X, I172N and P30L).

**Results:**

The 5 point mutations were identified in 31 of the 55 (56%) studied patients, 16/21 (76%) in the salt-wasting, 12/18 (67%) in the simple virilizing and 3/16 (19%) in the nonclassical form. The Intron 2 mutation was the most frequent, followed by G318X and 8 bp deletion. Compound heterozygotes were found in 10 patients, all corresponded to classic forms of the disease.

**Conclusions:**

The causal *CYP21A2* gene mutation was detected in 56% (72% in classic CAH), which makes the method encouraging. The most frequent mutations observed were Intron 2 and G318X. The detection of mutations offers confirmation of diagnosis, prediction of phenotype and genetic counseling.

## Background

Congenital adrenal hyperplasia is an autosomal recessive group of diseases. 21-Hydroxylase deficiency (21OHD) is the most frequent cause of CAH accounting for 95–99% of cases [1–3], and is the result of mutations in the *CYP21A2* gene [4]. The *CYP21A2* gene is located in the highly polymorphic region of the major histocompatibility complex (HLA), on the short arm of chromosome 6, locus p21.3, accompanied by a *CYP21P* pseudogene, with which it has a 98% homology. Moreover, they are located in tandem after the 3 ‘terminal portion of the 2 genes that code for the fourth complement component (C4A and C4B) [2, 4].

Like all recessive diseases, patients with CAH have both alleles (maternal and paternal) affected, and only 1% is the result of a spontaneous mutation. Gene abnormalities are variable and include anything from point mutations to large deletions. The clinical phenotype is the result of the combination of these abnormalities in the two *CYP21A2* alleles with the least affected allele usually determining the phenotype [3‚ 4].

Molecular analysis of the *CYP21A2* gene in patients with 21OHD, in North American and European populations, has shown that about 25% of these patients present macrodeletions of approximately 30 Kb, which include not only much of the 5′ region of the *CYP21A2* gene, but also all of the C4B gene and 3′ region of the *CYP21P* gene, or *CYP21A2* gene macroconversions in a similar way to *CYP21P*. The remaining 75% have gene microconversions or point mutations (PM) [5, 6]. The most frequent PM described in the classical forms are: I172N, R356W and G318X, a change from C to G in intron 2 which produces a splicing acceptor site 12 bases upstream of the normal splicing acceptor site, called ln2, a deletion of 8 bp in exon 3 that generates a shift in the reading frame and the appearance of a premature stop codon, called Ex3, and three T substitutions for A in codons 234–238, that determine a change in the sequence of Lle-Val-Glu-Met amino acids by Asn-Glu-Glu-Lys, called Cluster Ex6 [6, 7]. In the non-classic (NC) form, the most frequent PM described are P30L and V281L, respectively [8, 9].

On the other hand, deletions of the *CYP21P* gene associated with deletions of the *C4A* or *B* gene have been described in the normal population [8] and duplications of the *CYP21P* and *C4B* gene are frequently associated with NC 21OHD. Given the high homology between the *CYP21* genes and the complexity of the gene locus, the study at the molecular level is difficult. Thus, it is necessary to implement appropriate methodologies and strategies.

*CYP21A2* gene mutations have been studied in different ethnic groups, and it has been shown that a small number of mutations is responsible for the majority of 21OHD cases [8–11]. Thus, identifying only these few mutations can be used in a simplified and cheaper *CYP21A2* mutation analysis which may be more accessible in places with economical restrictions.

In 2005, the neonatal screening program was established in Cuba [12, 13], using 17-hydroxyprogesterone (17OHP) detection in dry blood on filter paper through the neonatal UMELISA Kit [14]. Its main objectives are early diagnosis of the classic form in both genders, to prevent life-treating salt-wasting crisis and to avoid incorrect sex assignment in the newborn.

The implementation of neonatal screening was undoubtedly an important step in the diagnosis and monitoring of patients with CAH in Cuba. However, only the clinical and biochemical diagnosis was available; the frequency of the different *CYP21A2* mutations in our population was not known, making prenatal diagnosis and personalized genetic counseling difficult.

The *CYP21A2* gene analysis is a useful complement to predict phenotype as well as confirming the diagnosis [15]. It is also important in situations where the 17OHP concentrations are unclear and in prenatal diagnosis when a child in a family is affected. Genotype may also predict long-term outcomes [16–18].

Thus, the aim of the current study was to characterize the genotype in patients diagnosed clinically with 21OHD and identifying the most frequent mutations in the Cuban population.

## Methods

A cross-sectional descriptive study was performed of all patients diagnosed clinically with 21OHD from January 2000 to December 2018 at the National Institute of Endocrinology, Havana, Cuba. The local ethical committee approved the study and informed consents were obtained.

For the molecular analysis of the *CYP21A2* gene, a protocol designed and approved by the National Center for Medical Genetics was used. The first stage used a 2-phase Polymerase Chain Reaction (PCR) and in the second stage, 5 different point mutations were detected (Intron 2, 8 bp Deletion, G318X, I172N and P30L). Characteristics of the mutational analysis in the *CYP21A2* gene are shown in Table 1.

**Table 1:** Characteristics of the mutational analysis in the *CYP21A2* gene

Mutation	Primers	Product of PCR (bp)	Restriction enzyme	Normal	Mutated
**Intron 2**	**P7 P8**	**378**	**HhaI**	**378**	**24, 354**
**Pro-30-Leu**	**P5 P6**	**249**	**HhaI**	**21, 228**	**249**
**I172N**	**P11P2**		**Taq I**	**416**	**394**
**Deletion 8-bp**	**P9 P10**	**89**	**–**	**89**	**81**
**Gln-318-Stop**	**P12 P13**	**136**	**PstI**	**25, 111**	**136**

Statistical analysis was performed using the SPSS program (version 19). Frequency distributions of qualitative variables were obtained, as well as mean (or median) and standard deviation (or interquartile range) according to whether the distribution was normal (or not). A *p*-value of less than 0.05 was considered statistically significant.

## Results

A total of 55 patients underwent *CYP21A2* gene mutation analysis to determine the presence of five common point mutations and these were identified in 31 patients (56%), of which 28/38 (74%) with classic forms and 3/16 (19%) NC form. Table 2 present the clinical characteristics of the phenotype groups.

**Table 2:** Manifestations and clinical signs in 55 studied patients with 21-hydroxylase deficiency

Phenotype	Salt-wasting (***n*** = 21)	Simple virilizing (***n*** = 18)	Non-classic (***n*** = 16)
Age of diagnosis	13.4 ± 6.3 days	12.8 ± 3.4 months	13.6 ± 3.7 years
Gender (n)	18F/3 M	10F/8M	15 F/1 M
Hyponatremia and hyperkalemia at presentation (n)	14F/1 M	0	0
Neonatal virilization (n)	17F	8F	0
Macrogenitosomy (n)	2 M	4 M	1 M
Scrotal hyperpigmentation (n)	3 M	7 M	1 M
Bone age accelerated (n)	2F	2F/1 M	3F
Early pubarche (n)	0	3F	3F
Hirsutism (n)	0	0	8F
Precocious pseudo-puberty (n)	0	2F/1 M	0
Tall stature (n)	0	5F/1 M	0
Acne (n)	0	1F	2F
Menstrual disorders (n)	1F	1F	6F

*F* female, *M* Male

In relation to the familial reproduction history, 23 (42%) identified some pathological elements, 10 (19%) infertility, 9 (16%) had been referred for spontaneous abortions, 7 (13%) hirsutism, 5 (9%) had a history of known CAH diagnosis in first-degree relatives, 2 (4%) neonatal death of unknown cause, 2 (4%) polycystic ovary syndrome and 1 (2%) atypical genitalia of unknown cause. No consanguinity was present. When the studied patients were analyzed in detail, 22 (71%) detected 1 mutation, 5 (16%) 2 mutations, 3 (10%) 3 mutations and 1 (3%) 4 of the investigated mutations. The details of the five point mutations studied are specified in Table 3.

**Table 3:** Frequency of 5 different point mutations found in the three phenotypes of patients with 21-hydroxylase deficiency in the Cuban population

Point mutations	Clinical forms of CAH			
	Salt-wasting	Simple virilizing	Non-classical	Total
Homo Intron 2	4	2	–	6
Homo G318X	1	1	–	2
Homo I172N	1	–	–	1
Hetero Intron 2	2	3	1	6
Hetero G318X	4	1	2	7
Hetero I172N	–	–	–	–
Homo I172N Hetero Intron 2, p30L and 8pb	1	–	–	1
Homo P30L and 8pb	1	–	–	1
Hetero Intron2p30L and 8pb	2	–	–	2
Homo Intron2 Hetero P30L and 8pb	–	1	–	1
Hetero G318X and I172N	–	1	–	2
Homo Intron2 and Hetero G318X	–	1	–	1
Hetero Intron2 and I172N	–	–	–	–
Hetero Intron2 and G318X	–	1	–	1
Hetero Intron2 and 8pb	–	1	–	1
Total	16	12	3	31

*Homo* Homozygous, *Hetero* Heterozygous

**Fig. 1:**
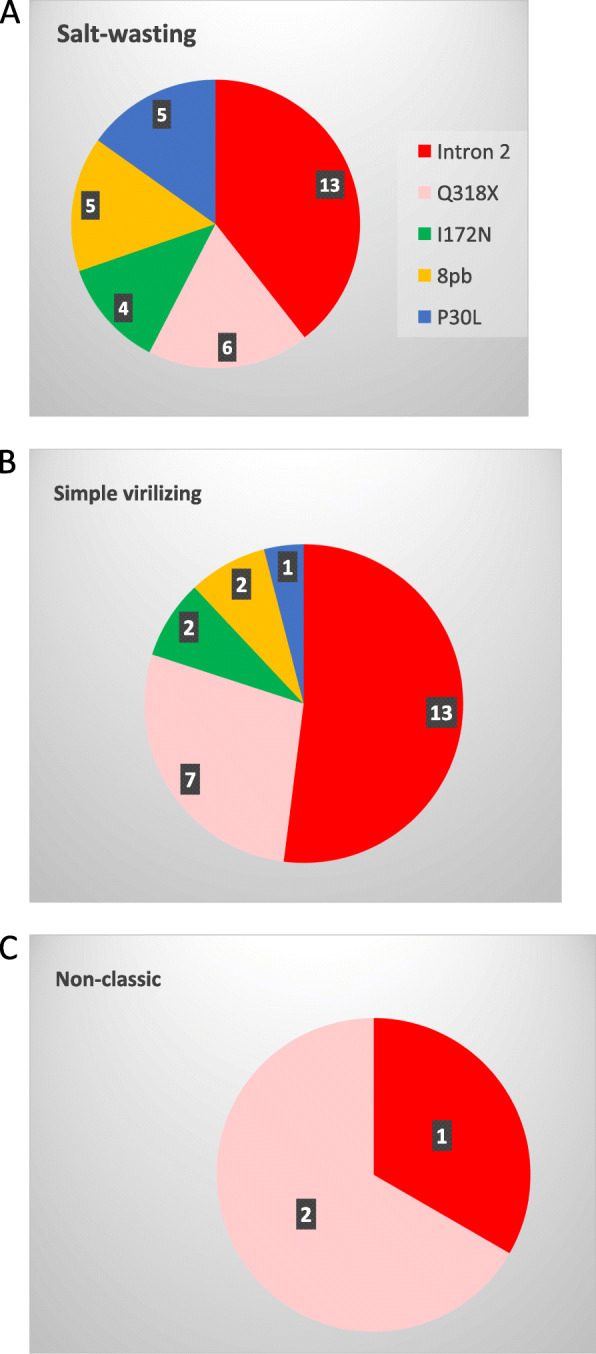
Distribution of affected alleles according to point mutations found in each clinical phenotype. Panel **a** Patients with salt-wasting phenotype. Panel **b** Patients with simple virilizing phenotype. Panel **c** Patients with nonclassical phenotype

**Fig. 2:**
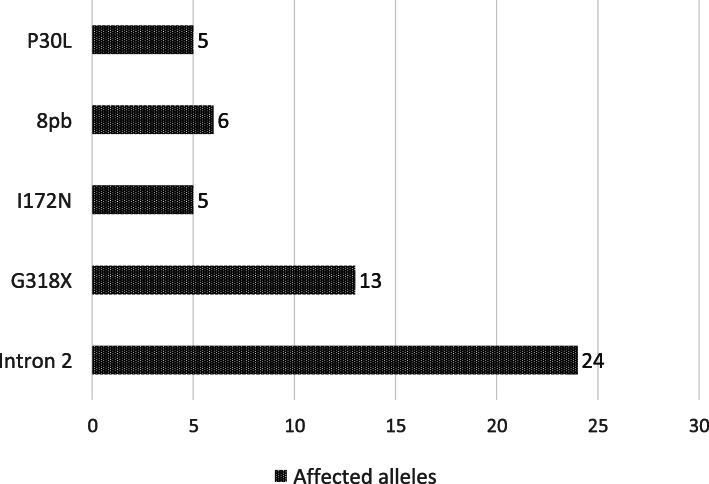
Affected alleles according to the five explored point mutations

The most frequent PM in the SW forms was Intron 2 (9/15, 60%), 4 of them in homozygosis, 2 in heterozygosity and 3 in compound heterozygosity with other mutations (Homo I172N Hetero Intron 2, P30L and 8pb (1) and Hetero Intron 2 P30L and 8 bp (2)). Also in the SV form Intron 2 mutation was the most frequent (9/11, 82%), present in 3 of those in homozygous, 3 in heterozygosis and the rest in compound heterozygosity (Hetero Intron 2 and I172N (1), Hetero Intron 2 and G318X (1) and Hetero Intron 2 and 8 bp (1)). Finally, in the NC form (all diagnosed with the ACTH stimulation test with measurements of 17OHP levels) the causal mutation was identified in 3/16 patients (19%), 2 heterozygous for G318X and 1 heterozygous for Intron 2. Figure 1 shows the distribution of the identified mutations according to the number of alleles affected, in each of the clinical presentation forms. The most frequent mutation was Intron 2, followed by G318X, deletion of 8 base pairs, and I172N and P30L (Fig. 2).

The presence of single chromosome abnormalities in a group of patients suggest that family members should be studied. Due to laboratory limitations, only in 10 families both parents were studied and in 4 more families only one parent (Table 3). When analyzing the 10 heterozygous patients, girls predominated (8/10) and 4 of the patients presented with classic forms (Table 4). According to the genotype, they were distributed into three groups, of which G318X was present in 7 of 10 patients (70%). The segregation of the mutation was 50/50 maternal/paternal.

**Table 4:** Analysis of heterozygous patients with 21-hydroxylase deficiency and their families

Genotype	Sex	Transmission/ Mutation	Clinical expression	Age at diagnosis
Intron 2, Del 8pb and P30L	M^a^	Maternal	Virilization of external genitalia Polyuria/Polydipsia. Insipidus diabetes Dehydration episode	2 years
Intron 2	F	Maternal	Virilization of external genitalia	30 days
Hetero G318X and I172N	F	Maternal/ Heterozygous I172N Paternal Heterozygous G318X	Virilization of external genitalia	3 years
Hetero Intron 2 and 8pb	M	Maternal/ Heterozygous Intron 2 Paternal Heterozygous P30L	Macrogenitosomy	3 years
G318X and I172N	M	Maternal	Macrogenitosomy Scrotal hyperpigmentation	41 days
G318X	F	Paternal	Virilization of external genitalia	First year
G318X	F	Maternal	Early adrenarche	6 years
G318X	F	Maternal	Dehydration episode Virilization of external genitalia	7 days
G318X	F	Paternal	Precocious pubarche	5 years
G318X	F	Paternal	Precocious pubarche	6 years

^a^ 46XX karyotype, assigned as male from birth. *F* Female, *M* Male

## Discussion

This study is the first report regarding the main *CYP21A2* mutations causing CAH due to 21OHD in the Cuban population. Of the five point mutations investigated, at least one were identified in 31 of the 55 (56%) studied patients with clinical diagnosed 21OHD. The Intron 2 mutation was the most frequent, followed by G318X and 8 bp deletion.

CAH is the most important cause of 46,XX disorder of sexual development (DSD), constituting 87.5% of all 46,XX DSD in Cuba [19, 20]. Newborns screening for CAH in Cuba [13] was an important milestone for the early hormonal diagnosis but genetical diagnosis was still an unsolved problem. Molecular genetic studies in CAH started many years ago [9]. In general, genetic studies are very expensive, which is a limitation for developing countries, such as Cuba. Based on studies on ancestry in the Cuban population [20–24], it has been estimated that our population received a contribution of genes of European origin (72–83%), African origin (13.8–26%) and Native American origin (0.8–3.2%). These results served as a basis for reviewing the frequency of PMs identified in the Spanish population [25] and those recognized with the highest prevalence were chosen to be explore in the present investigation.

Currently, direct analysis of the gene encoding the enzyme 21-hydroxylase is possible, and the detection of gene alterations causing the disease. However, as some studies have shown [26], direct analysis can sometimes provide limited information due to the presence of complicated rearrangements between *CYP21A2* and *CYP21P*, which make it difficult to determine whether the individual is affected or not.

More than 200 mutations have been described, PMs, small deletions, insertions and complex rearrangements of the gene. The most common mutation appears as a result of one or two types of meiotic recombination events between *CYP21A2* and *CYP21P*: 1) misalignment and uneven crossing, resulting in large DNA deletion, and 2) gene conversion events. Apparently, they give rise to the transfer to *CYP21A2* of small mutations present in the pseudogene [27, 28].

Throughout the years, the different methodologies used to study the *CYP21A2* gene have increased our genetic knowledge of 21OHD [4, 9, 29]. It has been possible to find characteristic mutations in some populations [29], including unaffected individuals [30]. Therefore, molecular analysis of the *CYP21A2* gene is the key to understand the etiology of 21OHD, both in basic science and in clinical diagnosis.

The economic cost of such genetic studies is a limitation for our country; hence, it was only possible to study the presence of five frequent PMs. The strategy used in this study offered encouraging results; with causal mutation being detected in more than half of the affected patients. In particular, in the group of SW patients, mutations causing severe deficit were identified in the majority. It was striking that not all of them had severe mutations in both the paternal and maternal chromosomes. In addition, in some of them, several mutations were observed on the same chromosome, so the total number of mutations detected was higher than that of the unrelated chromosomes studied. For this reason, it is important to carry out, in all cases, both the detection of large rearrangements using the Southern blot technique, and the widest possible analysis of mutations by PCR, to be able to establish an exact genotype.

The most frequent point mutation in the SW form of the Cuban population studied was the intron 2 mutation, which coincides with other studies [27, 31]. The severe G318X mutation was the second most common mutation, similar to that observed in other populations [32–34]. In addition, the 8 base pair deletion in exon 3 was present in 3 of the SW patients, in all, in compound heterozygosity, and more frequent than others have described [30]. In all, 76% of the SW cases were characterized genetically.

Moreover, this limited methodology allowed the characterization of 12 of 18 patients (67%) with the SV form. The frequency of mutations in this phenotype was somewhat peculiar, the frequency of the Intron 2 mutation was present in 64%, much higher than that found in other populations [26, 33, 35]. In all homozygous Intron 2 patients in our study, 4 of them had the SW form and the other 3 the SV form. In some patients it may exist in a certain degree, correct processing of the messenger ribonucleic acid (RNA) [36]. The rest of the patients with SV phenotype presented a similar mutation in compound heterozygosity.

Unlike other studies [31, 37] where the P30L mutation was found with high frequency in patients with the NC form, in our population P30L was only found in 3 patients with classic form, all in compound heterozygosity, which is rare.

Regarding patients with the NC form, molecular genetic analysis allowed only the characterization in about 20% of patients. It is possible that the remaining patients had other mutations not measured in this study or new mutations that do not exist in the pseudogene. The most common mutation in the NC phenotype is V281L [38], and unfortunately, we were not able to analyze this PM. Hence, new studies to further characterize this group in our population is needed.

Of the 3 patients with NC forms, 2 presented the G318X mutation in a heterozygous state and 1 was heterozygous for Intron 2. On the other hand, the P30L mutation, which is described as frequent in NC phenotype, was not found [27, 36]. It should be noted that the 3 patients characterized presented severe mutations in heterozygosity, causing classical forms, which would not have been possible to detect without using these molecular techniques and, moreover, they pose important implications for genetic counseling in these families [39]. Considering that it is an autosomal recessive hereditary disease, it is expected that patients with enzyme deficiency, regardless of their clinical form, presented abnormalities in the 21-hydroxylase gene on both chromosomes.

However, when the entire sample of patients was analyzed, it was observed that contrary to expectations, 17 of the patients only presented *CYP21A2* gene mutations in 1 of their chromosomes, which obviously complicates performing reliable prenatal diagnosis in this group. When jointly analyzing heterozygous patients, we did not find any common feature that could facilitate prenatal diagnosis. The predominance of girls and the large number of patients with the G318X mutation should be noted.

## Conclusions

The causal *CYP21A2* gene mutation was detected in 56% (74% in classic CAH), which makes the method encouraging. The most frequent mutations observed were Intron 2 and G318X. The detection of mutations offers confirmation of diagnosis, prediction of phenotype and an personalized genetic counseling.

## Data Availability

The datasets generated and/or analyzed during the current study are not publicly available, it belongs to the National Institute of Endocrinology. They are available from the corresponding author on reasonable request.

## Abbreviations

CAH: Congenital adrenal hyperplasia
21OHD: 21-Hydroxylase deficiency
DNA: Deoxyribunucleic acid
HLA: Major histocompatibility complex
C4A and C4B: Fourth complement component
PM: Point mutation
SW: Salt-wasting
SV: Simple virilizing
NC: Non- classical
PCR: Polymerase Chain Reaction
DSD: Disorder of sexual development
RNA: Ribonucleic acid
